# A comparison of glycosaminoglycan distributions, keratan sulphate sulphation patterns and collagen fibril architecture from central to peripheral regions of the bovine cornea

**DOI:** 10.1016/j.matbio.2014.06.004

**Published:** 2014-09

**Authors:** Leona T.Y. Ho, Anthony M. Harris, Hidetoshi Tanioka, Naoto Yagi, Shigeru Kinoshita, Bruce Caterson, Andrew J. Quantock, Robert D. Young, Keith M. Meek

**Affiliations:** a Structural Biophysics Group, School of Optometry and Vision Sciences, Cardiff Centre for Vision Sciences, Cardiff University, Wales, United Kingdom; b Connective Tissue Biology Laboratories, School of Biosciences, Cardiff University, Wales, United Kingdom; c Department of Ophthalmology, Kyoto Prefectural University of Medicine, Kawaramachi dori, Kamigyo-Ku, Kyoto, Japan; d Japan Synchrotron Radiation Research Institute, Spring-8, Sayo, 1-1-1 Kouto, Hyogo, Japan

**Keywords:** Cornea, Proteoglycans, Glycosaminoglycans, Collagen structure

## Abstract

This study investigated changes in collagen fibril architecture and the sulphation status of keratan sulphate (KS) glycosaminoglycan (GAG) epitopes from central to peripheral corneal regions. Freshly excised adult bovine corneal tissue was examined as a function of radial position from the centre of the cornea outwards. Corneal thickness, tissue hydration, hydroxyproline content, and the total amount of sulphated GAG were all measured. High and low-sulphated epitopes of keratan sulphate were studied by immunohistochemistry and quantified by ELISA. Chondroitin sulphate (CS) and dermatan sulphate (DS) distributions were observed by immunohistochemistry following specific enzyme digestions. Electron microscopy and X-ray fibre diffraction were used to ascertain collagen fibril architecture. The bovine cornea was 1021 ± 5.42 μm thick at its outer periphery, defined as 9–12 mm from the corneal centre, compared to 844 ± 8.10 μm at the centre. The outer periphery of the cornea was marginally, but not significantly, more hydrated than the centre (H = 4.3 vs. H = 3.7), and was more abundant in hydroxyproline (0.12 vs. 0.06 mg/mg dry weight of cornea). DMMB assays indicated no change in the total amount of sulphated GAG across the cornea. Immunohistochemistry revealed the presence of both high- and low-sulphated epitopes of KS, as well as DS, throughout the cornea, and CS only in the peripheral cornea before the limbus. Quantification by ELISA, disclosed that although both high- and low-sulphated KS remained constant throughout stromal depth at different radial positions, high-sulphated epitopes remained constant from the corneal centre to outer-periphery, whereas low-sulphated epitopes increased significantly. Both small angle X-ray diffraction and TEM analysis revealed that collagen fibril diameter remained relatively constant until the outer periphery was reached, after which fibrils became more widely spaced (from small angle x-ray diffraction analysis) and of larger diameter as they approached the sclera. Depth-profiled synchrotron microbeam analyses showed that, at different radial positions from the corneal centre outwards, fibril diameter was greater superficially than in deeper stromal regions. The interfibrillar spacing was also higher at mid-depth in the stroma than it was in anterior and posterior stromal regions. Collagen fibrils in the bovine cornea exhibited a fairly consistent spacing and diameter from the corneal centre to the 12 mm radial position, after which a significant increase was seen. While the constancy of the overall sulphation levels of proteoglycans in the cornea may correlate with the fibrillar architecture, there was no correlation between the latter and the distribution of low-sulphated KS.

## Highlights

• Proteoglycans (KS, DS, CS) and collagen were correlated with corneal radial position.
• Total sulfate levels on glycosaminoglycans remained constant across the cornea.
• KS and DS were ubiquitous; CS was found towards the edge of the cornea onwards.
• High-sulfated KS remained constant; low-sulfated KS increased peripherally.
• There was no correlation between fibrillar architecture and sulfation levels of KS.

## 1 Introduction

Unlike other collagen-rich connective tissues, the cornea transmits light in the visible part of the spectrum. This property is believed to be the result of collagen fibrils in the corneal stroma which are uniformly thin in diameter (approximately 30 nm in man (Meek and Leonard, 1993)) and are arranged with short-range spatial order (Maurice, 1957, Benedek, 1971). The uniformity in size of stromal collagen fibrils is thought to be influenced by associated intrafibrillar and/or extrafibrillar collagen molecules. For example, the increased presence of type V collagen molecules in the predominantly type I collagen fibrils is believed to limit fibril diameter (Birk et al., 1990, Birk, 2001). In addition, other abundant components within the corneal extracellular matrix, small leucine rich proteoglycans (SLR PGs), are also believed to have functional significance, with regulatory effects on diameter, spacing and specific spatial organization of collagen fibrils (Maurice, 1957, Benedek, 1971, Borcherding et al., 1975, Hassell et al., 1983, Vogel et al., 1984, Rada et al., 1993, Schönherr et al., 1995). Cornea is unique because of the presence of most of the different SLR PGs within one tissue, including four containing keratan sulphate. The ordered structure of the corneal stroma is essential for transparency and it is therefore subject to relatively strict positional regulation of its components, in contrast to fibrous tendon or the opaque sclera, which contain fewer PGs (Scott, 1995). These specific properties of the cornea make it a useful model for relating the subtle intricacies of the tissue to its specific functions and hence studying the various structural roles of sulphated proteoglycans found in connective tissues generally.

PGs are characterized depending upon the nature of their glycosaminoglycan (GAG) chains. GAGs are linear chains of repeating disaccharides that occupy a large volume of space and exhibit swelling pressure at relatively low concentrations (Scott, 1995). The abundance in sulphation of the amino sugars in the GAG chain confers a high negative charge to the unbranched molecule, which is important for stromal hydration and interactions with other PGs, both of which can influence the collagen architecture (Bettelheim and Plessy, 1975, Scott, 2001). In cornea, there are two main types of GAGs: chondroitin sulphate/dermatan sulphate (CS/DS) and keratan sulphate (KS), the latter being predominantly found in the adult human corneal stroma (Suzuki, 1939, Funderburgh, 2000, Quantock et al., 2010). These GAG chains are covalently bound to various core proteins to form proteoglycans, with lumican, keratocan, fibromodulin and mimecan bearing keratan sulphate GAG chains and decorin and biglycan possessing chondroitin sulphate/dermatan sulphate side chains (Blochberger et al., 1992, Li et al., 1992, Corpuz et al., 1996, Funderburgh et al., 1997, Funderburgh et al., 1998, Liu et al., 2003, Koga et al., 2005, Chen et al., 2010). Collagen fibrillogenesis studies have shown that the core protein of decorin, with or without its GAG side chain(s), can restrict the diameter to which collagen fibrils grow, and the lumican proteoglycan can act in a similar manner (Rada et al., 1993, Raspanti et al., 2008). Lumican knockout mice (in which keratocan is also affected (Carlson et al., 2005)) and decorin/biglycan knockout mice display pockets of larger-than-normal corneal collagen fibrils with irregular profiles (Chakravarti et al., 1998, Chakravarti et al., 2000, Zhang et al., 2009). The ratio of KS:CS/DS is greater in corneas of animals which have thick corneas than in animals with thin corneas, thought to be due to the high presence of over-sulphated terminal domain of KS (Scott and Bosworth, 1990); thus, the total content of sulphated GAG is relatively constant across species (Scott and Bosworth, 1990). As hypothesized by Scott and associates (Stockwell and Scott, 1965, Scott et al., 1989), this may be a consequence of the fact that the synthesis of KS requires no oxygen, while CS is synthesized in the presence of oxygen. Therefore, the production of KS would be favored in thick corneas, especially in deeper stromal regions (Bettelheim and Goetz, 1976, Castoro et al., 1988). Furthermore, the distribution of CS/DS and KS from the corneal centre to the periphery, in human, is more uniform, changing only at the limbus where KS decreases in quantity relative to DS (Borcherding et al., 1975). In the present study, we investigated collagen fibril parameters (fibril diameter and interfibrillar spacing) and sulphation patterns of KS GAG across the cornea from the centre to the periphery, to test whether different stromal KS GAG patterns are associated with altered fibril structure and organization. The distributions of CS/DS GAGs were also observed for comparison.

## 2 Results

### 2.1 Biochemical analysis across the bovine cornea

**Fig. 1:**
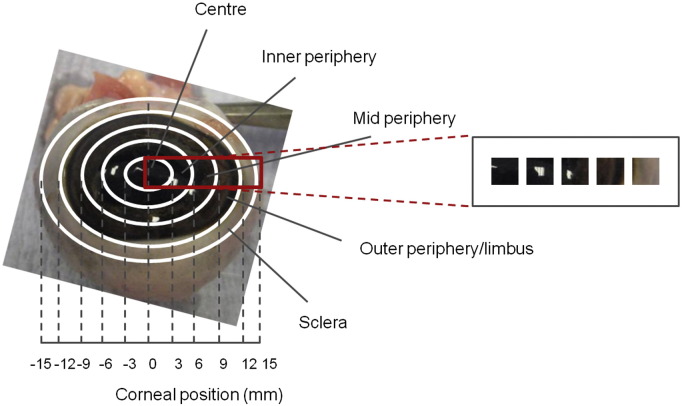
Schematic showing sites across bovine cornea and adjacent sclera investigated in this study. The red box represents the corneal strip that was excised, showing the sampling locations, designated: centre, inner periphery, mid periphery, outer periphery/limbus and sclera which were isolated for analysis.

**Fig. 2:**
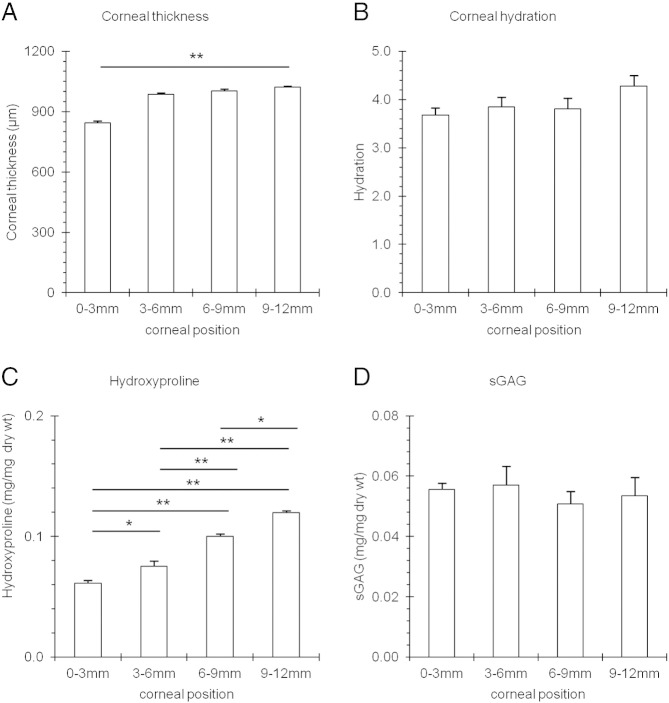
Corneal thickness (A.), hydration (B.), hydroxypoline content (C.) and total amount of sulphated GAG (D.) across the bovine cornea as a function of position. 0–3 mm = centre, 3–6 mm = inner periphery, 6–9 mm = mid periphery, 9–12 mm outer periphery/limbus. One-way ANOVA and post-hoc Tukey HSD tests were employed to identify the significant differences between the groups. (*) *p* = ≤ 0.01. (**) *p* = ≤ 0.001.

Measurements were made from different radial positions across the bovine cornea (Fig. 1) as described in detail in the Experimental Procedures section. Ultrasonic pachymetry of whole eyes revealed that the corneal thickness increased from 844.70 ± 8.10 μm in the 3 mm diameter central region to 1021.00 ± 5.42 μm in the limbal region, outside a radial distance of 12 mm (*p* ≤ 0.01) (Fig. 2A). The hydration across the bovine cornea also increased, from H = 3.67 ± 0.50 in the central 3 mm region to H = 4.28 ± 0.71 in the outer periphery; however, this increase was not statistically significant (*p* = 0.171) (Fig. 2B). The amount of hydroxyproline present in the centre of the bovine cornea, when expressed as a proportion of the dry weight of the tissue sample (0.06 ± 0.01 mg/mg dry wt), was doubled at the outer periphery (0.12 ± 0.01 mg/mg dry wt) (Fig. 2C). Interestingly, the DMMB analysis revealed no significant alteration in the amount of total sulphated GAG across the cornea from its centre to edge, which may indicate that the sulphation of the GAG population as a whole, i.e. KS and CS/DS, remains constant (Fig. 2D).

### 2.2 KS sulphation distribution across the bovine cornea

**Fig. 3:**
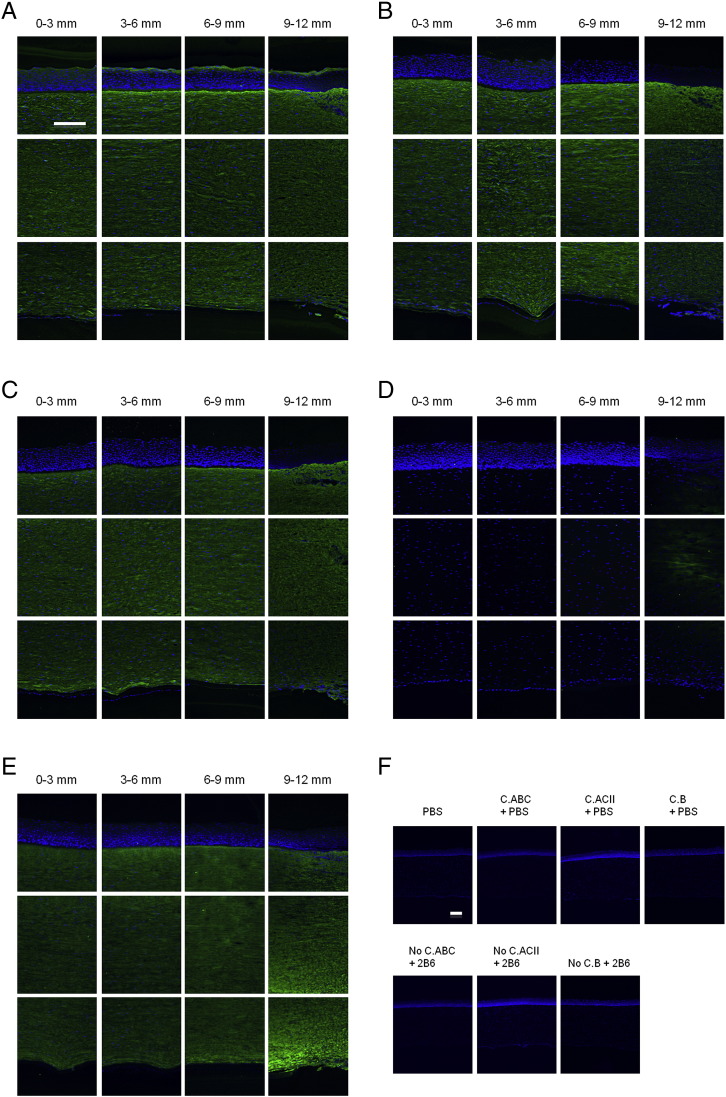
Immunolocalization of GAGs across bovine corneal tissue. (A) High-sulphated KS and, (B) lesser-sulphated KS were labelled with 5D4 and 1B4 antibodies, respectively. 2B6 antibody was used to specifically recognize: (C) DS GAG chains after pre-treatment with chondroitinase B; (D) CS GAG chains following pre-digestion with chondroitinase ACII; or, (E) CS/DS GAG chains after pre-digestion with chondroitinase ABC. Controls for the three antibodies, with/without enzyme pre-treatments showed negative staining (F). 0–3 mm = centre, 3–6 mm = inner periphery, 6–9 mm = mid periphery, and 9–12 mm = outer periphery/limbus. Scale bar = 200 μm.

By immunohistochemistry, high-sulphated KS identified by the 5D4 monoclonal antibody appeared to be uniformly distributed as a function of tissue depth, and this was the case from the corneal centre to periphery (Fig. 3A). Immunolabelling for lesser-sulphated KS using the 1B4 antibody was also qualitatively uniform throughout the full corneal thickness at all radial positions (Fig. 3B). In addition, uniformity of labelling was also seen with the 2B6 antibody, detecting DS GAG epitopes after pre-treatment with chondroitinase B (Fig. 3C). Interestingly, after pre-treatment with chondrotinase ACII to enable CS detection with 2B6 antibody, labelling was only detected towards the outer periphery–limbus region (Fig. 3D). Chondroitinase ABC exposed both CS and DS epitopes, and immunolabelling was therefore seen throughout the cornea (Fig. 3E).

**Fig. 4:**
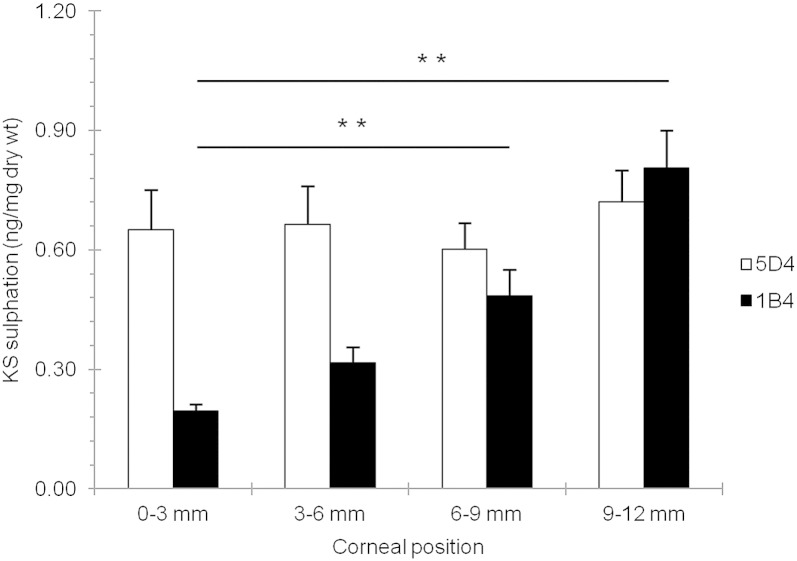
KS GAG sulphation content across the bovine cornea. 5D4 antibody was used to label high-sulphated KS GAG chains; with 1B4 antibody used to label lesser-sulphated KS GAG chains. 0–3 mm = centre, 3–6 mm = inner periphery, 6–9 mm = mid periphery, and 9–12 mm = outer periphery/limbus. One-way ANOVA and post-hoc Tukey HSD tests were employed to identify the significant differences between the groups. (*) *p* ≤ 0.01.

To investigate the KS immunolocalization observations in more detail, high and low sulphated KS epitopes in digested corneal tissue were quantified using a series of ELISAs. The results indicated that the amount of 5D4-recognizible high-sulphated KS remained relatively constant between the central 3 mm of the cornea (0.65 ± 0.31 ng/mg dry wt) and the outer periphery at a 12–15 mm radial location (0.70 ± 0.30 ng/mg dry wt; *p* = 0.290) (Fig. 4). However, the amount of lesser-sulphated, 1B4-recognizible KS epitope increased several fold in the outer peripheral cornea (0.8 ± 0.3 ng/mg dry wt) compared to the central region (0.2 ± 0.1 ng/mg dry wt; *p* = ≤ 0.001).

### 2.3 Collagen distribution across and through the depth of the bovine cornea

**Fig. 5:**
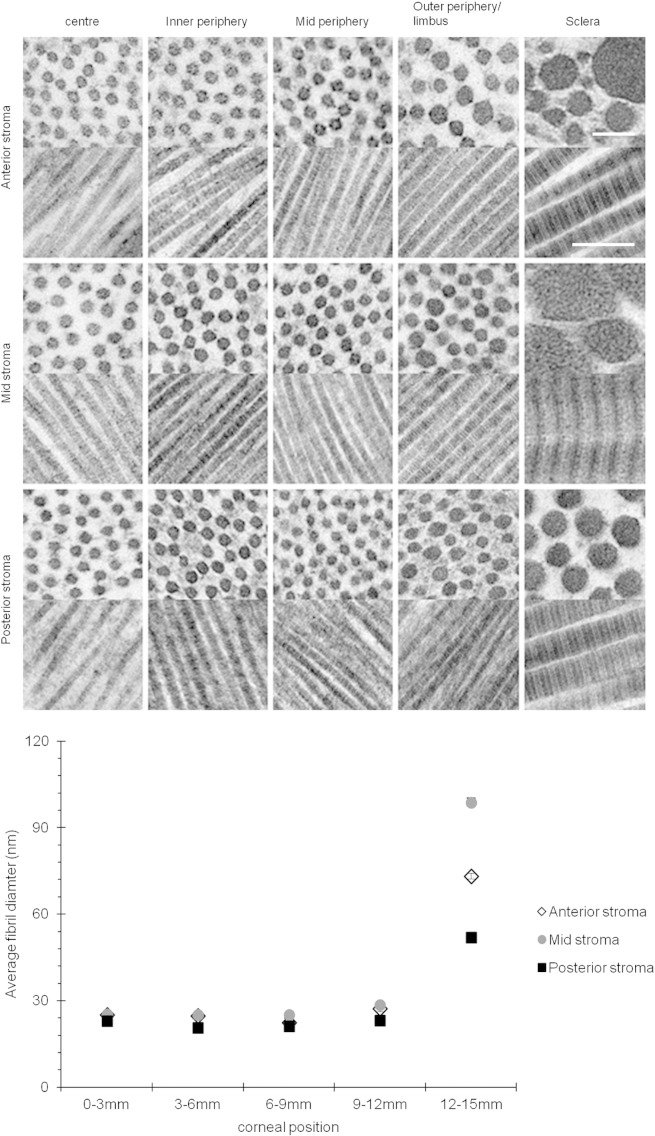
Transmission electron microscopy of collagen fibrils in transverse and longitudinal section across the bovine cornea. Scale bar = 100 nm (transverse section) and 200 nm (longitudinal). A summary of the mean fibril diameter across the bovine cornea is shown in the graph. 0–3 mm = centre, 3–6 mm = inner periphery, 6–9 mm mid periphery, 9–12 mm = outer periphery/limbus and 12–15 mm = sclera.

Transmission electron micrographs at sites in anterior, mid and posterior stroma (Fig. 5) show that collagen fibril diameter remained relatively uniform from mid cornea outwards to the mid-peripheral region (centre = 25.07 ± 0.09 nm, mid periphery = 22.35 ± 0.08 nm). In the outer peripheral/limbal region, fibrils in the anterior stroma varied in diameter with an overall increased average (27.28 ± 0.19 nm). At tissue locations more than 12 mm from the corneal centre into the anterior sclera much larger fibrils were evident (73.20 ± 0.96 nm).

**Fig. 6:**
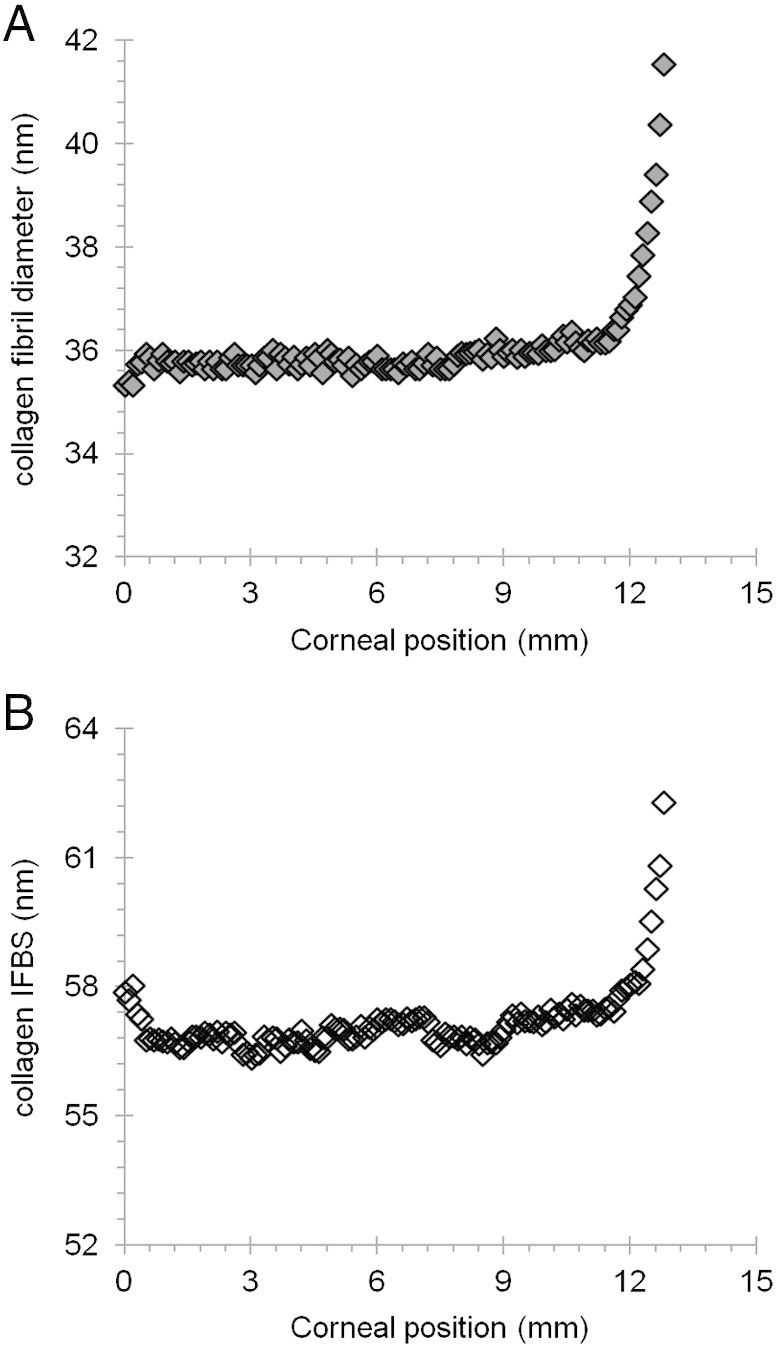
Collagen fibril diameter (A.) and interfibrillar Bragg spacing (IFBS) (B.) measurements across the bovine cornea.

**Fig. 7:**
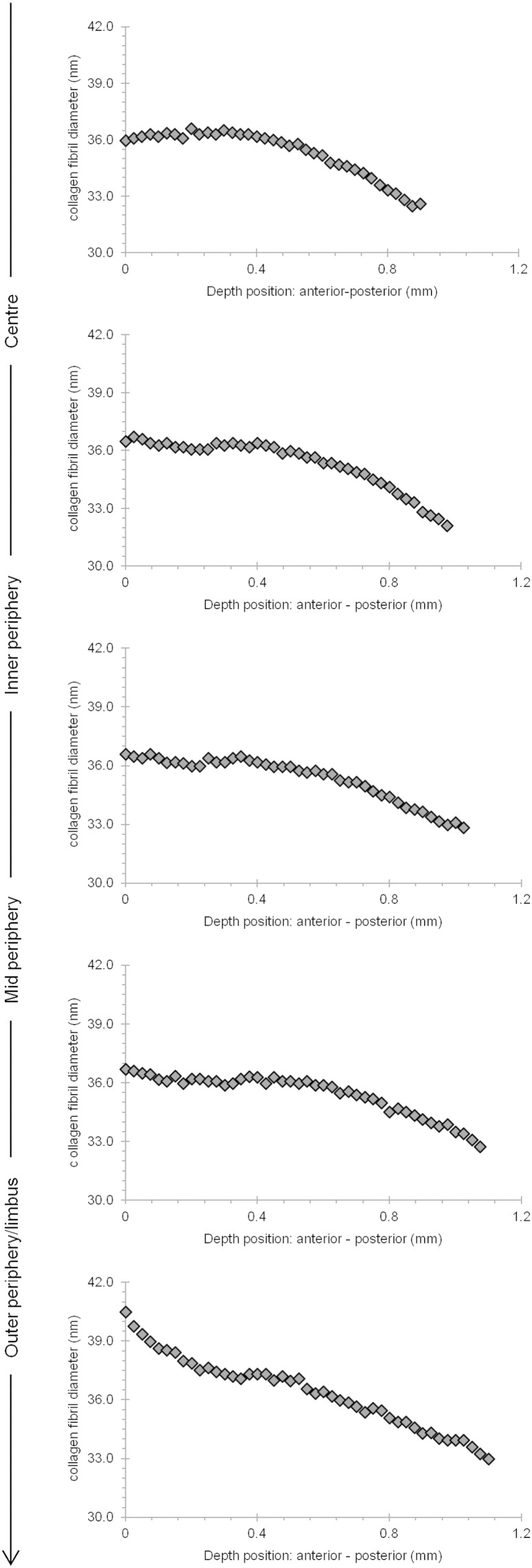
Collagen diameter as a function of tissue depth at different sites across the bovine cornea.

**Fig. 8:**
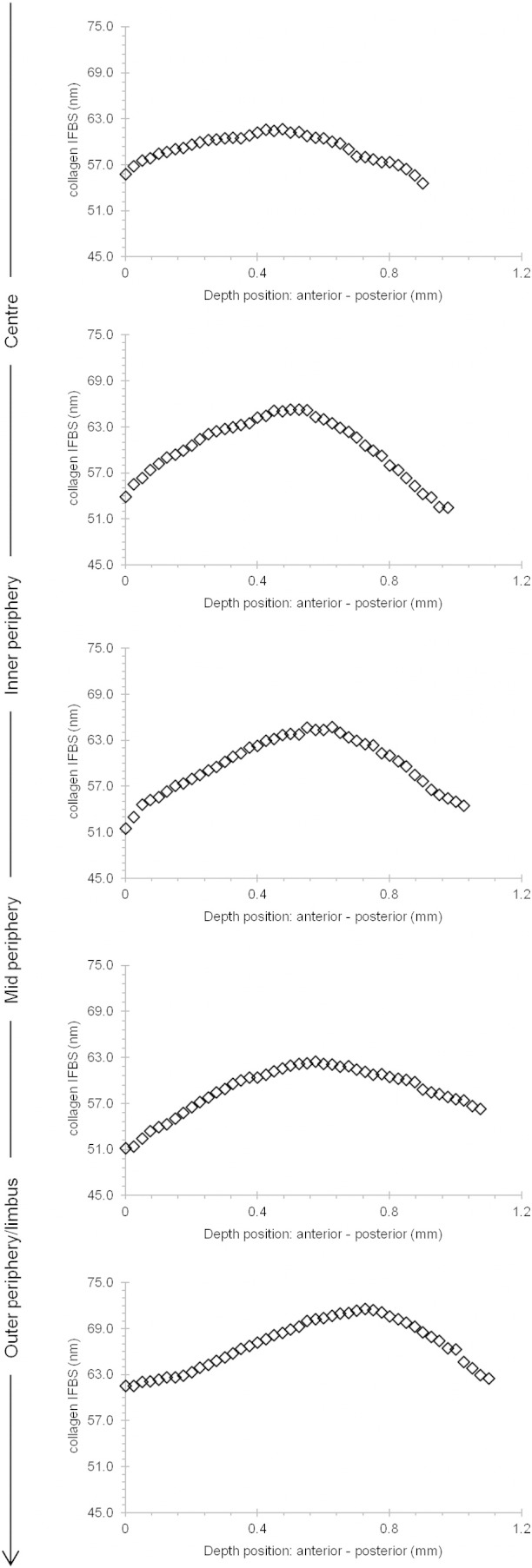
Collagen interfibrillar Bragg spacing (IFBS) as a function of tissue depth at different sites across the bovine cornea.

X-ray diffraction data provide excellent average information since all collagen fibrils in the path of the incident X-ray beam contribute to the diffraction pattern. This analysis revealed that from the centre of the cornea to the 9–12 mm outer–peripheral zone, the collagen fibril diameter remained relatively constant (Fig. 6A). Past the limbus into the sclera, as expected, the change was dramatic and collagen fibrils were of a much larger diameter. The X-ray diffraction analysis also indicated that the average centre-to-centre collagen fibril Bragg spacing remained constant from the centre of the cornea to the periphery of the cornea (Fig. 6B). Interestingly, the depth-profiled X-ray diffraction study of the collagen ultrastructure disclosed that the average diameter of collagen fibrils in the bovine cornea gradually decreased from the anterior regions of the cornea to deeper ones. The pattern was of a reduction in fibril diameter of between 9 and 12% between the superficial sub-epithelial stroma and the deep supra-Descemet's zone, and this was fairly consistent in the central cornea, the inner periphery, mid periphery and outer periphery to a 12 mm radial position. Moreover, there was an impression that collagen fibril diameter remained constant until about 40–50% of the stromal depth and dropped thereafter (Fig. 7). An interesting trend with stromal depth was also found in measurements of average collagen interfibrillar Bragg spacing, because at all radial positions the fibril spacing was higher at mid-stromal depth than at more anterior or posterior regions of the stroma (Fig. 8). The general trend throughout the whole cornea inside the 12 mm radial zone was for a fairly symmetrical change in collagen fibril spacing around a peak at approximately mid stromal depth; in the centre of the cornea, fibril spacing increased by 8% from the sub-epithelial zone to the mid stroma and then decreased by 9% towards the deep pre-Descemet's tissue. In the inner periphery, as an example, these percentage values were 18% and 19%, respectively, with the pattern of higher collagen fibril spacing in the mid stroma maintained across the bovine cornea.

## 3 Discussion

It has long been known that the cornea thickens away from the central optical zone. However, it was not clear if the thickening was due to increased stromal hydration, more collagen, or a combination of both. Bearing in mind the documented problems when attempting to measure hydration from small corneal samples (Doughty et al., 2001) our results indicate that there appears to be no statistically significant changes in hydration across the bovine cornea. There does, however, appear to be an increase in collagen content, strongly suggesting that the increased thickness arises from additional lamellae in the peripheral cornea, many of which may have their origins in the neighbouring limbus or sclera (Aghamohammadzadeh et al., 2004).

Structurally, the corneal extracellular matrix is thought to be governed, in part, by interactions between collagen fibrils and PG molecules modified with sulphated GAG side chains. Maintaining the characteristic stromal architecture is important to facilitate light transmission and corneal transparency (Maurice, 1957, Benedek, 1971). Light transmission through the human and bovine cornea is lower in the corneal periphery than in the centre, both in the visible spectrum and in the UV (Doutch et al., 2008, Doutch et al., 2012). Doutch et al. (2008) modelled this lowering of transparency peripherally, and partially explained it by the fact that collagen fibril diameters increased, moving from central to peripheral human cornea as reported by Boote et al., 2003, Boote et al., 2011. However, it was necessary to make the assumption that there were no significant proteoglycan matrix changes that would cause the refractive index of the interfibrillar space to change from the centre to the periphery of the stroma. The current results have supported this assumption. In the human cornea, the increased corneal thickness towards the periphery was reasoned to have only a minor effect on transmission. The data presented here indicate that in bovine cornea there is a similar peripheral increase in collagen fibril diameter as was reported in the human cornea (Boote et al., 2003, Boote et al., 2011), as well as a thickened cornea with more collagen mass, as evidenced by hydroxyproline quantification. It seems, therefore, that despite being proportionately larger, the bovine cornea, when moving from the centre to the edge, exhibits similar optical and structural modifications to the human cornea.

Much evidence using animal knockout studies has shown that PGs play a vital role in collagen fibril arrangement and orientation (Chakravarti et al., 1998, Chakravarti et al., 2000, Liu et al., 2003, Rühland et al., 2007, Zhang et al., 2009, Chen et al., 2010). GAG components of PGs allow them to act as “spacers” between fibrils. The sulphation patterns of the GAG components on PGs contribute to this because of the electrostatic fields they induce which permit their aggregation and dissociation. Using specific enzyme digestion, specific GAG chains could be visualized by electron tomography, revealing that PGs with CS/DS chains form longer aggregates that extend across several collagen fibrils, whereas the KS PGs are shorter and connect adjacent fibrils (Lewis et al., 2010, Parfitt et al., 2010). It could be the case that both types of GAG chains regulate the interfibrillar spacing through stabilizing adjacent fibrils, forming multimers and regulating the swelling pressure of the tissue through their sulphate residues, thus facilitating further packing in order to maintain the corneal collagen fibril architecture (Bettelheim and Plessy, 1975).

The early study by Borcherding et al. (1975) showed that KS is predominantly found in central human cornea and gradually depletes towards the limbus–sclera regions, but it still remains to be explained why the cornea, uniquely, contains four separate KS PGs, and what their individual roles might be. Interestingly, sulphated chondroitin was mainly found towards the peripheral cornea and into the sclera, while DS was only found in the limbus and sclera regions. Our immunostaining images showed a different pattern, such that KS and DS were found throughout the corneal stroma and into the sclera, while CS was only detected towards the outer periphery of the cornea onwards. Species differences may explain these variations as well as the technique used to distinguish CS and DS GAG chains. Although, further studies are needed, the location of specific PGs has given us some clues. For example, decorin is widely expressed, closely interacts with type I collagen and has been considered to influence the kinetics of collagen fibrillogenesis by primarily regulating lateral fibril growth and the distance between fibrils (Schönherr et al., 1995, Zhang et al., 2009). In corneas of decorin/biglycan-null mice, severe disruption in fibril structure and organization, especially at the posterior corneal regions, was seen (Zhang et al., 2009), indicating that CS/DS PGs may have a crucial role in the posterior regions of the cornea. Lumican shares certain functions in common with decorin, such as an involvement in many cellular processes: cell proliferation, migration, ECM metabolism, protein synthesis and fibrillogenesis (Rada et al., 1993, Cornuet et al., 1994, Doane et al., 1996, Saika et al., 2000). Abundant evidence has demonstrated the severity of lumican knockout in mice, with substantially altered collagenous matrix characterized by heterogeneous fibril diameters and disorganized fibril spacing, leading to the formation of cloudy corneas (Birk and Trelstad, 1984, Chakravarti et al., 1998, Chakravarti et al., 2000). With such major disturbance to the matrix structure in the absence of either lumican or decorin/biglycan in the cornea, we would speculate that both PGs would be present throughout the cornea to maintain normal fibril architecture by regulating fibril assembly.

Unlike lumican, keratocan, with sulphated KS chains, is limited to the corneal stroma in adult tissues, and the expression of this proteoglycan is considered a phenotypic marker for keratocytes (Carlson et al., 2005). Mutations of the keratocan gene and defective synthesis of its KS chain have been shown to cause flattening of the overall curvature of the cornea and blindness in humans by disrupting the organization of collagen fibrils (Liu et al., 2003, Carlson et al., 2005). Furthermore, keratocan shares a high degree of similarity in amino acid sequence, post-translational modification, and corneal localization with lumican and thus, from the current evidence, it could be speculated that keratocan may function in a similar manner to lumican, but strictly within the corneal stroma. Carlson et al. (2005) indicated that keratocan may be regulated by lumican and may serve as a downstream regulator of the overall architectural frame of the cornea (Carlson et al., 2005).

Fibromodulin knockout mice share similar collagen matrix abnormalities with lumican knockouts in connective tissues, including cornea, namely a high degree of small and large diameter fibrils during collagen fibril formation and maturation (Chakravarti et al., 1998, Chakravarti et al., 2000, Chen et al., 2010). Therefore fibromodulin may have a similar functional role to lumican, but relating more specifically towards the limbus and sclera, where it becomes restricted during development. It has been suggested that fibromodulin may act to stabilize small-diameter fibril-intermediates in the cornea, while lumican may be needed at a later stage, primarily to limit lateral growth of fibrils. Fibromodulin may thus play an important role in regulating region-specific fibrillogenesis such as that required at the limbus for the integration of corneal and scleral matrices.

Previous biochemical and histochemical studies of cartilage (Stockwell and Scott, 1965) and cornea (Scott and Haigh, 1988) have pointed to an inverse relationship between tissue thickness and KS content, which has led to the concept that KS is the GAG synthesized preferentially in conditions of low O_2_ supply (Scott and Haigh, 1988). Indeed, studies have shown a gradient of KS content throughout the depth of the cornea, with KS increasing posteriorly (Bettelheim and Goetz, 1976), which follows the decline of O_2_ tension across the depth of the cornea. Thus, because the bulk of the O_2_ supplied to the cornea comes via the ocular surface, the deep stromal layers of thick corneas of large animals such as humans or ox are believed to experience relatively low levels of O_2_ and preferentially express high levels of KS. This is not the case in the thin cornea of the mouse, on the other hand, which has unusually low levels of sulphated KS throughout its whole depth (Young et al., 2005). Our immunostaining images, however, do not show a clear differential in staining density of KS signal throughout the depth of the cornea, so perhaps any difference is below the sensitivity of the immunolabelling approach.

Depth profile studies (Quantock et al., 2007) of human eye-bank corneas have shown that there are no significant changes in collagen fibril diameter throughout the central region of the tissue, although a lower collagen interfibrillar spacing in the anterior-most stromal regions was found, compared with the spacing in the deeper cornea. The differences with the current investigation could be due to species variation, or the fact that in the earlier study (Quantock et al., 2007) swollen human corneas were used and it is known that fibril spacing in the cornea is sensitive to the tissue's water content (Meek et al., 1991, Fratzl and Daxer, 1993). Our data, however, showed that in bovine corneas (Fig. 6, Fig. 7) fibril diameters were reduced with depth, particularly in the posterior layers of the cornea. Interfibrillar Bragg spacing through the depth of the cornea showed the same trend at all positions across the tissue, initially increasing to a depth of about 61–65 μm then falling off in the posterior 52–57 μm of the cornea (Fig. 8). Lower values in anterior and posterior areas may have been due to the loss of water from the surfaces while the cornea was being cut for the depth-profile experiments. However, this was unlikely, as the average interfibrillar spacing from the sections was similar to that from the intact tissue.

In summary, thickening of the cornea at its periphery is mirrored by elevated levels of hydroxyproline in this region of the tissue, indicative of more collagen. Across most of the cornea the overall level of sulphated GAG—i.e. KS and CS/DS—remains steady, as does the average diameter of the collagen fibrils. The tissue content of sulphated KS from the central to the peripheral regions of the bovine cornea reported here follows a similar trend to that reported by Borcherding et al. (1975) in their experiments on human cornea. Those authors discovered that KS is found throughout the cornea, but reduced towards the corneo-limbus and beyond. Our study expands on this, and shows that high-sulphated KS is present throughout the bovine cornea from the centre to the periphery (Borcherding et al., 1975). We also provide evidence that lesser-sulphated epitopes of KS are present in the corneal centre, and that the amount of these epitopes increases, steadily and linearly through the inner-periphery, mid periphery and outer periphery to the 12 mm radial position. This increase in low-sulphated KS towards the corneal periphery does not correlate with any change in the collagen fibril structure; thus, any structural significance of this regional change in KS GAG composition is unclear. Further studies are needed to define the roles of sulphated KS PGs, as well as those of CS and DS, in relation to their distribution across the cornea. Furthermore, the fact that average collagen fibril spacing and diameter does not change appreciably across the bovine cornea suggests that the role of the high-sulphated KS GAGs, potentially aligned to the actions of other corneal PGs, is sufficient to exert a controlling influence on the fibril architecture.

## 4 Experimental procedures

Whole bovine eyes were obtained from a commercial abattoir a few hours after slaughter and stored on ice. Unlike the human eye (Boote et al., 2003), no accepted terminology exists for the different regions across the bovine cornea, so in the current study, the bovine cornea was categorized into 3 × 3 mm squares from the central zone and out towards the sclera, as indicated in Fig. 1.

### 4.1 Corneal thickness measurements

Eyes with visibly clear corneas (*n* = 10) had their corneal thickness measured using a DGH Technology Inc Ultrasonic Pachymeter. Corneal thickness was recorded as an average of 15 readings at each tissue region (centre (0–3 mm zone), mid-periphery (3–6 mm zone), periphery (6–9 mm zone) and outer periphery (9–12 mm zone)).

### 4.2 Hydration measurements

Fresh whole corneas (*n* = 10) were excised (epithelial and endothelial cells removed), after which a 3 mm-wide horizontal strip was cut across the central diameter of each cornea. Whole-thickness pieces of tissue were then dissected to represent the central 3 mm-zone, and the more peripheral tissue regions in 3 mm steps from the centre of the cornea outwards as indicated in Fig. 1. Tissues were weighed, freeze dried, and weighed again, after which the hydration was calculated using, Eq. (1):

(1) $$\mathrm{Hydration}=\;\frac{\mathrm{wet}\;\mathrm{wt}–\mathrm{dry}\;\mathrm{wt}}{\mathrm{dry}\;\mathrm{wt}}$$

### 4.3 Sample preparation for biochemical studies

Ten sets of 3 mm × 3 mm corneal samples corresponding to the defined corneal regions were double extracted in 4 M guanidine HCl to remove PGs and non-collagenous proteins. The soluble (supernatant) and insoluble fractions were separated, after which the residue was twice extracted with 0.5 M acetic acid containing 0.5 mg pepsin per ml. The pepsin extracts were then pooled, freeze-dried and stored at 4 °C until needed. In addition, 10 sets of 3 mm × 3 mm corneal samples were digested with papain (1 mg/ml, Sigma-Aldrich, UK) in 0.05 M sodium acetate buffer (pH 5.6), containing 0.025 M EDTA and 5 mM cysteine HCl for 17 h at 60 °C. The enzyme was then inactivated at 100 °C, and digested samples were stored at − 20 °C until needed

### 4.4 Hydroxyproline content

The amount of collagen in dissected corneal pieces was quantified using a hydroxyproline assay (Blain et al., 2006). Papain digests from individual samples were hydrolysed by adding equal volumes of 11.7 N HCl to supernatant at 110 °C overnight. Specimens were then lyophilized. Dried hydrolysates were reconstituted in their starting volume of distilled water and centrifuged to remove particulate material. Hydroxyproline residues were assayed against known standards (L-hydroxyproline, Sigma-Aldrich, UK) and read at 540 nm after 10 min incubation at 70 °C.

### 4.5 DMMB assay

The sulphated GAG content in papain-digested samples was measured by colorimetric assay with dimethylmethylene blue (DMMB). Chondroitin sulphate C, supplied as the sodium salt from shark cartilage (Sigma-Aldrich, UK), was used as a standard (Farndale et al., 1986) and readings were taken at a wavelength of 525 nm.

### 4.6 Immunohistochemistry

Small, full-thickness samples of bovine cornea were cut from the central 0–3 mm zone, the 3–6 mm zone, the 6–9 mm zone, and the 9–12 mm zone and embedded in OCT. Cryosections, 8 μm thick, were blocked with an Image IT Signal Enhancer (Invitrogen, UK)/5% normal goat serum for 20 min then incubated with the anti-keratan sulphate primary antibodies 5D4 (1:100 final dilution) and 1B4 (1:50 final dilution). Additional sections were also incubated for 1–2 h at 37 °C with antibody, 2B6 (1:20 dilution)—specific for CS/DS GAGs, with or without enzyme pretreatment to expose the CS and DS epitopes: chondroitinase ABC (0.4 U/ml) for CS and DS GAGs, or chondroitinase ACII (0.1 U/ml) (Seikagaku Corp., Tokyo, Japan) for CS GAGs, or chondroitinase B (0.5 U/ml) (R & D Systems, Inc., MN) for DS GAGs. Sections were then labelled with Alexa Fluor 488-conjugated goat anti-mouse IgG secondary antibody, mounted with Prolong Gold containing the nuclear stain DAPI (Invitrogen, UK) and examined on an Olympus BX61 microscope and F-View digital camera.

### 4.7 KS quantification

Competitive ELISAs were used to quantify the pentasulphated hexasaccharides and tetrasulphated hexasaccharides in small linear KS chains, which were recognized by monoclonal antibodies 5D4 and 1B4, respectively (Caterson et al., 1983, Mehmet et al., 1986). 96 well microtitre plates were coated with 0.05 μg chondroitinase ABC (Sigma-Aldrich, UK) bovine corneal stroma antigen in 20 mM NaHCO_3_ buffer (pH 9.6), and incubated for 2 h at 37 °C, followed by an overnight incubation at 4 °C. The coated microtitre plates were washed with Tris saline azide (TSA) and the non-specific sites were blocked with the addition of 1% (*w/v*) bovine serum albumin (BSA) in TSA, and incubated for 2 h at 37 °C. After incubation, the blocked microtitre plates were washed, air dried, and stored at 4 °C until needed.

Papain digests of different corneal zones were serially diluted and allowed to bind with an equal volume of 5D4 (1:6000 final dilution in 1% BSA/TSA) or 1B4 (1: 150 final dilution in 1% BSA/TSA). A standard curve was generated from serial dilutions of chondroitinase ABC treated bovine corneal antigen/5D4 or 1B4, respectively. The plates were then washed with TSA before incubation with alkaline phosphatase-conjugated goat anti-mouse antibody (Promega, Madison, WI) used at 1:5000 dilution. The plates were again washed before alkaline phosphatase substrate (p-nitrophenylphosphate, 1 mg/ml) was applied in DEA buffer (0.126 mM MgCl_2_, 1 M diethanolamine, pH 9.8). Colour development was quantified on a plate reader (Multiskan MS; Labsystems, Helsinki, Finland) at 405 nm, to determine the inhibition of binding.

### 4.8 Transmission electron microscopy

For ultrastructural analysis, corneal tissue samples were isolated from respective radial positions, centre-to-periphery. Briefly, full-thickness cornea (2–3 mm scleral rim attached) was first fixed with 2.5% glutaraldehyde, 2% paraformaldehyde in 0.1 M Sorensen phosphate buffer, pH 7.4 at room temperature for 2 h. The tissue was then cut into sections according to the different positions and further fixed for 1 h at room temperature. Prior to post-fixation with 1% osmium tetroxide for 1 h, several washes in 0.1 M Sorensen phosphate buffer were applied. Samples were then contrasted *en bloc* in 0.5% uranyl acetate and subsequently dehydrated through an ascending ethanol series. After transferring the samples to propylene oxide, the samples were infiltrated and embedded in Araldite CY212 resin. Ultrathin sections (90–100 nm thick, cut with diamond knife) were collected on uncoated G300 copper grids, and stained with 1% aqueous phosphotungstic acid and uranyl acetate. Sections were examined in a transmission electron microscope (JEOL 1010; JEOL, Tokyo, Japan) equipped with a charge-coupled device camera (Orius SC1000; Gatan, Pleasanton, CA).

### 4.9 Small-angle X-ray diffraction

Fresh, clear bovine corneas were excised with a scleral rim, approximately 2–3 mm wide still attached, carefully wrapped in Clingfilm (Tesco, UK) to minimize dehydration, frozen to − 80 °C and transported on dry ice to the Spring-8 synchrotron facility in Hyogo Prefecture, Japan for X-ray fibre diffraction analysis.

On the high-flux beamline 40XU, the corneas were gently thawed and a 5 mm strip was cut horizontally across the central optical axis. The corneal strip was immediately placed between a single layer of Clingfilm to limit dehydration during exposure to the X-ray beam. The corneal strip was then mounted in the path of the beam that was focused to a diameter of 25 μm at the anterior surface of the cornea with the epithelium facing the X-ray beam. X-ray diffraction patterns were obtained in 100 μm steps from the corneal centre to its periphery. In a second experiment–—the depth-profiled experiment—a thin strip of cornea, again cut across the corneal diameter, was orientated so that the X-ray beam passed through the cut edge of the tissue in a direction parallel to the corneal surface. A series of X-ray diffraction patterns, again each 25 μm in diameter, was obtained from the epithelial to the endothelial surface of the corneal strip in 50 μm steps. This was done for every radial position (from epithelial to endothelial direction) from the centre to the outer periphery of the bovine cornea. Radiation of wavelength *λ* = 0.83 Å was used and diffraction patterns were recorded on 640 × 480 pixel detector with sub-second exposures. The 67 nm meridional reflection from hydrated rat-tail tendon was used as a calibrant, and the data was analysed to calculate average collagen fibril interfibrillar spacing and average collagen fibril diameter as described previously (Meek and Quantock, 2001).
